# Ethological and clinical evaluation of the welfare of horses in draft competitions in Spain

**DOI:** 10.3389/fvets.2026.1720309

**Published:** 2026-04-02

**Authors:** Marta E. Alonso-de-la-Varga, Juan Manuel Lomillos Pérez

**Affiliations:** 1 Departamento de Producción Animal, Facultad de Veterinaria, Universidad de León, León, Spain; 2 Departamento de Producción y Sanidad Animal, Salud Pública Veterinaria y Ciencia y Tecnología de los Alimentos, Facultad de Veterinaria, Universidad CEU Cardenal Herrera, Valencia, Spain

**Keywords:** animal welfare, clinical evaluation, draft competitions, ethology, horses

## Abstract

Draft and pulling horses are used in a traditional equestrian sport in the Valencian Community (Spain), in which horses must pull a cart loaded with a weight equivalent to twice their body weight along a sand track. Although it constitutes cultural heritage, this practice raises questions about animal welfare since it involves working horses under demanding physical exertion. There are no studies evaluating the welfare of horses participating in these draft and pulling competitions using validated clinical, ethological, and physiological indicators, and the present work aims to fill this knowledge gap. A total of 160 horses of different breeds and five weight categories were evaluated across five competitions. Health parameters, body condition, hoof status, presence of lesions, and behavioral responses to the observer were recorded. In 20 horses, heart rate was monitored using a heart rate monitor, and hematological analyses were performed. In addition, owners were surveyed regarding their social profile and management practices. Most horses showed an adequate body condition (74.4% scored 3/5) and a low incidence of harness-related injuries (≤15.6%), mainly superficial abrasions in older horses. Conformation defects were observed at 11.9% and hoof overgrowth at 19.4%. Regarding behavior, 65% of horses remained alert, and 68.8% responded amicably to the evaluator, while younger horses exhibited more avoidance and aggressiveness. During competition, heart rate peaked at 175 bpm, comparable to equestrian sports such as show jumping, with recovery to baseline values within 15 min; hematological analyses were normal in all cases. Overall, we conclude that draft pulling horses show good health and training status, with no evidence of serious welfare impairments. Nevertheless, strengthening hoof care and preventing harness-related injuries through training programs and regular veterinary check-ups is recommended.

## Introduction

The use of horses in draught and pulling competitions represents a deeply rooted traditional practice in regions such as the Valencian Community (Spain), where it constitutes a cultural expression of high heritage value and significant social impact (1, 2).

The sport of “tiro y arrastre” (draft horse pulling) consists of an exercise of endurance and intensity in which the horse pulls a cart weighing approximately twice its own body weight along a sand track (60 m long and 3 m wide), with three mandatory stops every 15 m. Different categories are defined according to the weight of the horse: 1st—up to 220 kg, 2nd—220–320 kg, 3rd—320–420 kg, 4th—420–520 kg, and 5th—over 520 kg (3).

The sport resembles the use of horses as working animals still prevalent in many developing countries (4–7) or other high-intensity equestrian disciplines (8), demands maximum physical effort from the horse, which may have consequences for its physical health, behavior, and emotional state.

Such competitions raise important questions regarding animal welfare, as equine are subjected to intense physical exertion under conditions that may not always align with their physiological and ethological capacities. Moreover, the increasing societal sensitivity toward the ethical treatment of animals has driven the need to establish scientific criteria to assess welfare in these types of competitions.

Frameworks such as the “Five Freedoms” (9) and the more recent “Five Domains” model (10) provide useful conceptual tools for the systematic evaluation of equine welfare. However, their application to the specific context of “tiro y arrastre” competitions requires contextual adaptation and scientific validation. Furthermore, it is crucial to consider the horse owners’ perspectives, as their beliefs, practices, and possible lack of knowledge may significantly influence welfare conditions (6, 11, 12), as human-animal interactions play and important role both in “Five Freedoms” (9) and “Five Domains” (10) welfare approaches.

Several factors affecting the welfare of draught horses have been described, including adequate nutrition, proper harnessing, veterinary care, and the development and promotion of programs aimed at raising social awareness of best management practices, among others (4, 7). Some studies also report physical and ethological health indicators based on responses to human presence and contact (5, 12–14), as well as physiological indicators (15–17).

For these reasons, a multidimensional approach to animal welfare is essential, encompassing physiological markers (such as heart rate, respiratory rate, and temperature), health indicators (body condition, pathologies, injuries), and ethological indicators (social behavior). Welfare state–based indicators are considered more reliable and relevant compared to resource-based measures (18–20).

This study aims to provide a comprehensive description of the welfare of horses participating in “tiro y arrastre” competitions by combining direct assessment of clinical, physiological, and behavioral indicators with information on environmental and management conditions provided by the owners. In addition to characterizing overall welfare status, the study seeks to explore potential interactions between welfare indicators, individual characteristics, including age and body weight, as well as management practices, with the purpose of identifying key risk factors and generating preliminary insights that may guide future hypothesis-driven research. This assessment will also support the proposal of evidence-based recommendations to ensure the welfare of these horses within the framework of this traditional equestrian sport.

## Materials and methods

The study was conducted over 6 months, from July to December 2022.

### Study area

This work was carried out in the Valencian Community (Spain), located on the eastern Mediterranean coast of Spain, encompasses approximately 23,255 km^2^ and exhibits a heterogeneous topography, consisting of narrow coastal plains and inland mountain ranges reaching elevations of up to 1,800 m. The region has a predominantly Mediterranean climate, with mild winters, hot dry summers, and low annual precipitation, although inland areas display continental and semi-arid variations. Its population is approximately 5.47 million inhabitants, largely concentrated in the coastal metropolitan areas of Valencia, Alicante, and Castellón.

**Figure 1:**
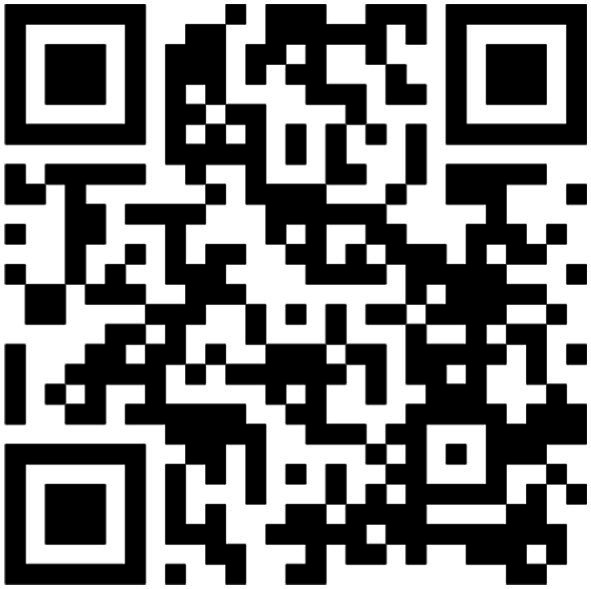
Video example of a “tiro y arrastre” competition (https://www.youtube.com/watch?v=QSZ4ib_rlHY).

The competitions evaluated in this study were held in five different municipalities within the region. All events followed the standard “tiro y arrastre” layout, consisting of a 60 m long and 3 m wide sand track, a surface chosen for its cushioning properties and reduced locomotor impact. Along the track, horses are required to perform three mandatory stops, spaced approximately every 15 m (Figure 1).

### Animals studied

A total of 160 horses of different breeds, weights, and ages were evaluated by three veterinarians specialized in animal health and production, all of whom have more than 10 years of experience as researchers and lecturers in the field. All horses participated in five competitions held in different municipalities of the study area and could be considered representative of those participating in this type of competition, as they were approximately 40% of the horses registered in the Draught Horses Federation.

“Tiro y arrastre” horses are typically maintained in small social groups of two to three individuals, allowing regular social contact. They generally have access to a shared outdoor paddock for free movement and interaction, while also being provided with individual indoor stalls that offer protection from weather conditions and a designated resting area. Their diet is primarily based on hay and other roughage sources, supplemented with commercial concentrate feed according to workload and body condition.

Most animals are acquired at approximately 15 months of age, and a gradual, progressive training process usually begins once they reach around 2.5 years of age. Training is commonly performed by the owners themselves and focuses on developing both physical conditioning and habituation to the equipment and competition environment (3).

Horses’ owners were informed beforehand about the purpose of the study, and each provided written informed consent and authorization for the use of the collected data. This form recorded the horse’s age, breed, sex, and identification through a RFID tagging.

All horses were transported to the competition venues on the same day of the event, with transport duration ranging from approximately 30 min to 2 h depending on origin. Upon arrival, animals were unloaded and allowed time to settle before evaluation and competition. Horses had free access to water both before and after their participation, and water was routinely offered again immediately after completing the trial. Unlike endurance or long-distance disciplines, “tiro y arrastre” competitions involve very short bouts of intense effort, with each pull lasting 60–90 s and total competition time per horse does not exceed 5 min, approximately (3). Under these conditions, sweat loss is minimal and does not justify routine electrolyte supplementation; consequently, salt pastes or electrolyte solutions are not commonly administered.

### Evaluation of animal welfare indicators

A structured protocol was developed to assess non-invasive animal welfare indicators, previously standardized by Pritchard et al. (19), validated and applied to draught horses by Romero et al. (13), to which some physiological indicators such as body temperature and heart and respiratory rates were added (15, 17).

The indicators consisted of a set of physical health and behavioral measures, based on responses to human presence and contact, following the work of Burn et al. (14) and Popescu et al. (5). Scoring one horse took approximately 30 min.

#### Behavioral indicators

Indicators related to behavioral parameters were recorded through four tests (Table 1):

- The general state of alertness of the horse was evaluated over 10 s at a distance of 6 m.
- At a distance of 3 m, the observer stood at an angle of approximately 20° (looking at the horse’s neck or chest, not directly in front), then approached to 30 cm from the horse’s head to record its response.
- The observer then walked alongside the horse from head to hindquarters, maintaining a distance of ~30 cm from its body, registering any evidence of alertness.
- The observer gently positioned one hand beneath the mandibular region of the horse, applying light contact sufficient to support a small amount of weight, but not enough to raise the head.

**Table 1:** Behavioral indicators related to equine welfare.

Recorded indicator	Categorizations	Description
General state of alertness	Alert	Ears moving forward, eyes open.
	Apathetic	Normal state.
	Depressed	Calm or with lowered head.
Response to the observer approached	Friendly	The horse turns its head toward the observer.
	Avoidance	The horse turns its head away, moves back, or lays its ears flat.
	Aggression	Attempts to bite or kick.
	No response	The horse does not respond.
Response to the observer walked alongside him	Friendly	Tail movement and return of the head toward the observer.
	Avoidance	The horse moves away.
	Aggression	Ear pinning, head turning, kicking.
	No response	The horse does not respond.
Response to gentle chin support without lifting	Avoidance	The horse moved its head away.
	Acceptance	The horse remained still.

Adapted from Pritchard et al. (19) and Romero et al. (13).

#### Health indicators

Indicators related to physical health were recorded wearing gloves following the methodology previously described (5, 13, 14), including body condition, temperature, heart and respiratory rates, coat and mucous membrane condition, vision, and hydration status. In addition, conformation and hoof condition were assessed. Finally, mobility was evaluated by asking the owner to walk the horse for approximately six straight-line steps, first away from and then back toward the observer, as a basic lameness examination (Table 2).

**Table 2:** Health indicators related to equine welfare.

Recorded indicator	Categorizations	Description
Body condition	1–5	Scale from 0 (very poor: ribs, backbone and hipbones prominent; topline of neck concave; pelvis hollow) to 5 (very good: backbone, pelvis and ribs not visible; topline of neck firm and convex; pelvis rounded with ‘gutter’ along spine)
Coat staring	Yes/no	Uneven coat, poor appearance, not shiny or glossy.
Ectoparasites	Present/absent	Presence of ectoparasites.
Dehydration	Yes/no	Skin tent (skin fold) <2 s (>3 s = mild to moderate dehydration).
Mucous membranas	Normal/abnormal	Moist gingival, labial, ocular mucosae with normal coloration or are pale, congestive, cyanotic or sticky, dry and dull.
Capillary refill time	Normal/abnormal	Refill time >2 s after pressing upper and lower gums (possible hypovolemia or dehydration).
Lesions at lip commissures	Present/absent	Visible skin or subcutaneous tissue lesions at the lip commissures, caused by bit.
Teeth missing	Yes/no	Incomplete or poor incisive dental condition.
Vision	Normal/abnormal	Menace response test by moving the hand slowly toward the eye from a lateral position (approximately 30–45°), starting at 30–40 cm and stopping at 5–10 cm from the cornea, while avoiding tactile contact, air currents, or auditory stimulation. Blinking and/or head withdrawal were considered normal responses.
Respiration	Normal/abnormal	Dilated nostrils, abnormal respiratory rate or depth, visible abdominal involvement in breathing.
Cough	Yes/no	Coughing episodes observed during evaluation.
Nasal discharge	Present/absent	Visible nasal discharge, except for occasional serous drops.
Ocular discharge	Present/absent	Presence of moist or dry ocular discharge; no visible residues on eyelashes or facial hair.
Skin lesions on the head associated with headstall use	Present/absent	No lesions >2 cm^2^ or >1 × 3 cm in headstall contact areas. Hair loss or simple scars not considered.
Hip lesions (iliac región)	Present/absent	No lesions >2 cm^2^ or >1 × 3 cm in harness contact areas. Hair loss or simple scars not considered.
Body lesions	Present/absent	Whole body assessment (excluding lips, harness points, and legs). Lesions larger than 2 cm^2^ or 1 × 3 cm should not be present.
Diarrheal signs	Present/absent	Loose feces, with signs of fresh or dried diarrhea under the tail.
Neck and chest lesions	Present/absent	Lesions caused by the harness collar.
Skin lesions on the thoracic region associated with girth use	Present/absent	Lesions caused by the girth.
Lameness	Present/absent	Signs of lameness in any limb.
Limbs lesions	Present/absent	Visible lesions on limbs (radiocarpal and tarsometatarsal regions).
Joints and tendons lesions	Present/absent	Visible swelling in tendons or joints inflammation (arthritis).
Hoof quality	Normal/abnormal	Hoof should be round, smooth, without cracks or missing fragments; straight shape in lateral view. No visible morphological defects in dorsal or lateral views.
Hoof growth	Normal/abnormal	Hoof wall excessively long or short.
Temperature	Normal/abnormal	Between 37.5°C and 38.5°C. >38.5°C = fever (infection, inflammation, heat stroke); <37.0°C = hypothermia (prolonged cold exposure or shock).
Resting heart rate	Normal/abnormal	Altered rate (>48 bpm) due to stress, pain, fever, anemia, dehydration, heart disease, respiratory failure, and toxemia.
Resting respiratory rate	Normal/abnormal	Altered rate (>20 rpm) due to stress, respiratory failure, fever, pain, acidosis, circulatory failure, and heart stroke.

Adapted from Popescu et al. (5) and Romero et al. (13).

Heart rate (HR) and respiratory rate (RR) data were manually recorded for all horses at rest, immediately before the competition, immediately after the trial, and 15 min post-trial.

After the previously mentioned data were registered and before the competition, blood samples were collected from 25 horses randomly selected from the 160 studied, ensuring representation of 5 animals per category, to assess for the assessment of their health status. The collection was done with the horse standing and its head gently restrained to ensure safety. After disinfecting the puncture site, the jugular vein was accessed using a sterile needle attached to a 10 mL vacuum tube containing EDTA, and the samples were subsequently sent to an accredited laboratory for analysis. A general hematological analysis was performed, evaluating various parameters that allow for assessment of overall health, detection of infectious or inflammatory processes, anemia, coagulation disorders, and other clinical conditions, including: red blood cell count, hemoglobin concentration, hematocrit, mean corpuscular volume, mean corpuscular hemoglobin, mean corpuscular hemoglobin concentration, red blood cell distribution width, white blood cell count, neutrophils, monocytes, eosinophils, basophils and their percentages, platelets, mean platelet volume, and platelet distribution width.

Environmental data (temperature and humidity) were recorded on the days of the competitions using a digital thermometer and hygrometer (PCE 555).

#### Horses’ owners information

Following the approach adopted in previous studies on sport horses (21–23), a structured questionnaire was administered to horse owners (*n* = 67) to collect information regarding their demographic characteristics, management and training practices. The questionnaire included items on owner age, sex, educational level, and place of residence, as well as number of horses owned, purchase source, and stable location. Additional questions addressed motivation for competition participation (e.g., family tradition), training frequency and duration, and whether training and daily care were performed personally or delegated to others. Finally, information about each horse’s age at purchase, number of training days per week, and competitions participated in was recorded. All owners signed a document agreeing to participate in the project with the anonymous information provided.

### Ethical statement

All procedures related to the use and care of animals followed strict protocols in accordance with current animal experimentation regulations (24) and were conducted with approval from the Animal Ethics Committee of CEU-UCH University (approval code: 2022 VSC PEA 0169). Additionally, data obtained from animal owners were collected and processed exclusively after obtaining their explicit informed consent to participate in the study, ensuring compliance with ethical standards for research involving human participants.

### Statistical analysis

Animals were treated as experimental units. Descriptive analyses were conducted for all measured variables and expressed as the proportion of horses exhibiting each behavioral or health parameter. Analyses involving age and body weight were conducted as exploratory comparisons, given the absence of previous data in this discipline. Following the methodology described by Pritchard et al. (19), related observations belonging to similar categories were combined into composite scores: (a) lack of responsiveness to environmental/handling stimuli (general attitude + response to observer approach + response to observer walking alongside), (b) poor body condition score (mucous membrane color + coat condition + diarrhea + dehydration), and (c) skin and deeper tissue lesions (friction injuries + swelling-related lesions + tendon or joint swelling + limb deformities + overgrown hooves + abnormal sole surface + hoof wall quality).

Associations between categorical welfare indicators, composite scores, and age or body weight categories were analyzed using Chi-square tests. Relationships between ordinal or non-normally distributed variables were evaluated using Spearman’s rank correlation coefficients.

Physiological variables, including heart rate (HR), respiratory rate (RR), and rectal temperature (T), were analyzed using analysis of variance (ANOVA) to assess differences between age and body weight categories.

Statistical significance was accepted at *p* < 0.05, while *p*-values between 0.05 and 0.10 were considered indicative of a trend. All analyses were conducted using IBM SPSS Statistics, version 30.0 (25).

## Results

### Characteristics of horses and breeders

Table 3 presents a description of the 160 horses sampled according to breed, age, and weight category in the competition.

**Table 3:** Distribution (% and total number) of evaluated horses (*n* = 160) among the different categories considered.

Classification variable	Category / Level	Category					Age			Total
		1	2	3	4	5	<5	5–15	>15	
Males	Intact male	6.87 (11)	8.75 (14)	15.63 (25)	16.25 (26)	13.13 (21)	11.88 (19)	39.38 (63)	11.25 (18)	60.63 (97)
	Gelding	10 (16)	6.88 (11)	10 (16)	5.63 (9)	6.88 (11)	3.13 (5)	18.13 (29)	16.25 (26)	39.38 (63)
	<5	1.25 (2)	0 (0)	5.63 (9)	3.75 (6)	4.38 (7)	–	–	–	15 (24)
Aged group (years)	5–15	10.63 (17)	12.50 (20)	13.75 (22)	12.5 (20)	8.13 (13)	–	–	–	57.50 (92)
	>15	5 (8)	3.13 (5)	6.25 (10)	5.63 (9)	7.50 (12)	–	–	–	27.50 (44)
Breed	Jaca Navarra	6.88 (11)	4.38 (7)	2.50 (4)	0 (0)	0 (0)	3.75 (6)	8.13 (13)	1.88 (3)	13.75 (22)
	Pottoka	5 (8)	3.13 (5)	0 (0)	0 (0)	0 (0)	1.88 (3)	5 (8)	1.25 (2)	8.13 (13)
	Caballo del Pirineo Catalán	0 (0)	0 (0)	7.50 (12)	3.75 (6)	0 (0)	2.50 (4)	5.63 (9)	3.13 (5)	11.25 (18)
	Hispano-Bretón	0 (0)	0 (0)	6.25 (10)	6.25 (10)	13.13 (21)	3.13 (5)	11.25 (18)	8.13 (15)	25.63 (41)
	Burguete	0 (0)	0 (0)	3.13 (5)	5.63 (9)	5 (8)	1.25 (2)	11.25 (18)	4.38 (7)	13.75 (22)
	Mixed-breed horses	5 (8)	8.13 (13)	6.25 (10)	6.25 (10)	1.88 (3)	2.50 (4)	16.25 (26)	8.75 (14)	27.5 (44)
Total		16.88 (27)	15.63 (25)	25.63 (41)	21.88 (35)	20 (32)	15 (24)	57.50 (92)	27.50 (44)	100 (160)

No mares were identified in the audited competitions, although it is possible that some mares may participate, but they are very rare. Regarding castration practices, the majority of horses were intact males. Horses were distributed across different age groups, with a predominance of horses aged 5–15 years, where the estimated average age was 11.7 years. The youngest horse was 3.5 years old and the oldest was 18 years old.

Many of the competing horses were crossbreeds, with the most common breed being the Hispano-Bretón, followed by Burguete, Jaca Navarra (79), Catalan Pyrenean, and Pottoka breeds, all classified as draft-type horses. Smaller breeds (Pottoka and Jaca Navarra) were mostly placed in categories 1 and 2, while the other three breeds (Burguete, Catalan Pyrenean, and Hispano-Bretón) were distributed in categories 3 and 4. Finally, category 5 was composed mostly of Hispano-Bretón horses.

The studied competitions were held under environmental conditions approaching the critical thresholds for heat stress in horses (26), with a mean temperature of 27.66 °C (range: 23.83–31.08 °C) and 69.34% humidity (range: 53.21–78.28%).

Regarding the owners, the information collected in the questionnaire to the horse owners is shown in Table 4. The owner usually represents a man of 56 years, living in rural areas, most of whom did not pursue higher education. On average, each breeder owned 2.4 horses housed in stables located within the horticultural production area in the eastern province of Valencia (Spain), near the Mediterranean Sea, where horses have traditionally been the main aid in agricultural work. The average training or work frequency was 3.81 days per week (each session lasting approximately 2.3 h), with horses participating in an average of 6.6 competitions per year.

**Table 4:** Results of the questionnaire completed by the owners (*n* = 67).

Category	Variable	Results
Owner demographics	Age	56.30 ± 19 years
	Sex	100% males
	Educational level	Primary 24 (35.80%)
		Secondary 28 (41.80%)
		University 15 (22.40%)
	Place of residence	Urban 11 (16.40%)
		Rural 56 (83.60%)
Horse ownership	Number of horses owned	2.4 horses
	Stable location	Same municipality 43 (64.20%)
		Different municipality 24 (35.80%)
	Reason for participation	Family tradition 57 (85.10%)
		Personal interest 10 (14.90%)
Training management	Training frequency	3.81 days per week
	Training duration	2.30 h per training session
	Responsible for training/care	Owner 37 (55.20%)
		Trainer 5 (7.50%)
		Shared 25 (37.30%)
	Number of competitions participated in a year	6.60 competitions
Veterinary care	Number of visits to the veterinarian per year	5.60 visits per year

### Evaluation of behavioral indicators

The majority of horses exhibited a response to their environment with a general alert attitude, with no significant differences (*p* > 0.05) between weight categories or age groups.

Similarly, a large proportion of horses displayed friendly behavior toward the evaluators, with slightly higher percentages of avoidance responses observed in category 1, and aggressive responses more common in horses from categories 1 and 2. However, these differences between categories were not statistically significant (*p* > 0.05).

On the other hand, significant differences (*p* < 0.05) were observed in the behavioral responses of younger horses, particularly in avoidance of contact with the observer and in aggressive responses.

### Evaluation of health indicators

The majority of horses had an adequate body condition, scoring 3 on a 1–5 scale. No horses were classified as having low (1) or very high (5) body condition. No significant differences were observed between weight categories or age groups (*p* > 0.05).

No cases of poor coat condition or ectoparasites were detected. Similarly, no signs of dehydration, abnormal mucous membranes, or abnormal capillary refill time were observed.

The head was observed to be in perfect condition: no alterations in the mouth (lip commissures and incisive teeth), no vision problems, and normal respiration without coughing or nasal/ocular discharge, with two horses with slightly elevated body temperatures. None of the horses showed signs of diarrhea.

No lameness or lesions were detected on the body or hips. Minor lesions from the headstall were observed in some horses on the head and lesions from the harness on the neck/chest, but not in the girth area (barrel). Conformational defects were present in some of horses. Mild joint inflammation was observed in three horses, 10 horses had poor hoof quality, and a notable percentage showed mild hoof overgrowth (Table 5).

**Table 5:** Frequency (%) and total number (*n*) of behavioral parameters observed in the evaluated equines (*n* = 160).

		Category					Age			*p*-value	*p*-value	Total
Category	Level	1	2	3	4	5	<5	5–15	>15	Cat.	Age	
*n*		27	25	41	35	32	24	92	44			
General attitude	Alert	70.37 (19)	68.0 (17)	51.22 (21)	65.71 (23)	75.0 (24)	79.17 (19)	64.13 (59)	59.09 (26)	0.26	0.244	65 (104)
	Apathetic/Depressed	29.63 (8)	32.0 (8)	48.78 (20)	34.29 (12)	25.0 (8)	20.83 (5)	35.87 (33)	40.91 (18)			35 (56)
Response to observer approach	No response	11.11 (3)	28 (7)	31.71 (13)	31.34 (11)	15.63 (5)	20.83 (5)^a^	26.09 (24)^a^	22.73 (10)^a^	0.12	<0.01	24.38 (39)
	Friendly response	70.37 (19)	60.0 (15)	65.85 (27)	62.86 (22)	84.38 (27)	50.0 (12)^a^	69.57 (64)^b^	77.27 (34)^b^			68.75 (110)
	Avoidance	11.1 (3)	4.0 (1)	0	2.86 (1)	0	12.50 (3)^a^	2.17 (2)^b^	0^b^			3.13 (5)
	Aggressive response	7.41 (2)	8.0 (2)	2.22 (1)	2.86 (1)	0	16.67 (4)^a^	2.17 (2)^b^	0^b^			3.75 (6)
Response to observer walking alongside	No response	7.41 (2)	20.0 (5)	31.71 (13)	22.86 (8)	18.75 (6)	20.83 (5)	20.65 (19)	22.73 (10)	0.052	0.22	21.25 (34)
	Friendly response	55.56 (15)	56.0 (14)	51.22 (21)	60.0 (21)	81.25 (26)	50.0 (12)	66.30 (61)	54.55 (24)			60.63 (97)
	Avoidance	25.93 (7)	8.0 (2)	9.76 (4)	8.57 (3)	0	12.5 (3)	7.61 (7)	13.64 (6)			10 (16)
	Aggressive response	11.11 (3)	16.0 (4)	7.32 (3)	8.57 (3)	0	16.67 (4)	5.43 (5)	9.09 (4)			8.13 (13)
Physical contact with observer	Accepts	37.04 (10)	40.0 (10)	53.66 (22)	54.29 (19)	71.88 (23)	12.5 (3)^a^	52.17 (48)^b^	75.0 (33)^c^	0.0601	<0.05	52.5 (84)
	Avoids	62.96 (17)	60.0 (15)	46.34 (19)	45.71 (16)	28.13 (9)	87.5 (21)^c^	47.83 (44)^b^	25.0 (11)^a^			47.5 (76)

Groups with different letters in red show significant differences (*p* < 0.05, post-hoc test).

Health indicators did not show significant differences by breed or weight category, although differences were observed according to age (*p* < 0.05) in friction-related skin lesions caused by headstall and harnesses, with a significantly higher incidence in horses older than 15 years. However, these lesions were few and mild, consisting of superficial rubs. Similarly, minor joint inflammation in three horses’ carpi or tarsi without signs of pain, no elevated temperature, and no signs of lameness.

Some horses exhibited poor hoof quality, with 19.38% showing mild overgrowth of the hoof wall, likely due to a lack of recent shoeing or trimming, with no significant differences between categories or ages. Additionally, the majority of horses were shoed.

Evaluation of conformation was challenging due to uneven ground; however, conformation defects were observed in 11.88% of horses, with no significant differences between groups. Most defects involved angular deviations in the forelimbs, with carpi (knees) deviated outward and hooves turned inward in frontal view (*n* = 8), commonly known as “estevados.” From the rear, some horses were observed with hocks too close together (“closed,”) or too far apart (“open,”), with hooves turned inward or outward. Two horses were observed with hindlimbs outside the vertical line of correct alignment.

A large percentage of horses exhibited increased heart rate (HR) and respiratory rate (RR), though not reaching pathological levels, with significantly higher values observed in younger horses (*p* < 0.01).

The aggregated behavioral score for the horse’s reaction to the observer was found to correlate significantly with both heart rate (HR) and respiratory rate (RR), and HR and RR were also significantly correlated with each other. Lesions on the skin caused by the harness and collar were significantly correlated with the aggregated score of limb problems (including conformation defects, arthritis, and poor hoof quality).

The mean duration of each horse’s participation in the trial was 65 ± 37 s, with no differences observed between weight categories, breeds, or age groups. Heart rate monitoring revealed the following pattern.

Temperature, heart rate, and respiratory rate measured before, immediately after, and 15 min post-trial (Table 6) showed significant differences between values recorded at the start and immediately after the trial, returning to values similar to baseline after 15 min.

**Table 6:** Mean ± standard deviation values temperature (T °C), heart rate (HR bpm) and respiratory rate (RR rpm) at rest, before, immediately after and 15 min post-trial.

Variable	Rest	Before trial	After trial	15 min post-trial	*p*-value
HR	39.18^a^ ± 12.52	45.18^a^ ± 23.73	175.24^b^ ± 21.01	58.21^a^ ± 13.02	<0.001
RR	29.82^a^ ± 10.68	38.58^a^ ± 12.09	63.24^b^ ± 23.11	38.81^a^ ± 14.11	<0.001
T	37.51^a^ ± 0.26	37.6^a^ ± 0.41	38.44^b^ ± 0.70	37.77^a^ ± 0.39	<0.05

Finally, hematological analyses were all within normal ranges, and all horses showed all parameters analyzed to be normal, with no significant differences (*p* > 0.05) between weight categories or age groups (Table 7).

**Table 7:** Mean and standard deviation of hematological values in sampled horses (*n* = 25) and reference values for the equine species (78).

Parameter	Mean (*n* = 25)	Standard deviation	Reference range
Erythrocytes (M/μL)	8.94	2.21	6.50–12.50
HCT (%)	42.91	8.96	32–52
HGB (g/dL)	14.54	3.39	11–19
MCV (fL)	48.50	2.44	37–55
MCH (pg)	16.51	0.64	15–20
MCHC (g/dL)	34.11	0.97	32–36
RDW (%)	16.98	2.05	14–18
Leukocytes (K/μL)	9.96	1.78	5.50–12.50
% Neutrophils	67.68	6.80	30–75
% Lymphocytes	28.83	3.97	25–60
% Monocytes	4.82	1.85	0–6
% Eosinophils	1.96	0.84	0–6
% Basophils	0.71	0.30	0–1
Neutrophils (K/μL)	6.75	1.44	2.50–8.50
Lymphocytes (K/μL)	2.46	0.73	1.50–5.50
Monocytes (K/μL)	0.49	0.23	0–0.60
Eosinophils (K/μL)	0.19	0.07	0–0.60
Basophils (K/μL)	0.07	0.03	0–0.15
Platelets (K/μL)	190.90	58.25	100–350
Mean platelet volume (fL)	7.67	0.45	6–11
Platelet distribution width (fL)	9.43	0.54	9–19
Thrombocrit (%)	0.14	0.03	0.10–0.30

## Discussion

### Characteristics of horses and owners

The majority of the sampled animals belonged to the 5–15 year age group, indicating that the studied population is primarily composed of adult horses of working or competitive age. The second most frequent group was those over 15 years old, while young horses (under 5 years old) were clearly in the minority.

This distribution shows that over 85% of the horses studied were over 5 years old, suggesting a predominantly mature population, consistent with the use of these animals in sporting or working activities (27). The low representation of young animals could be due to the fact that many are not yet fully integrated into training or competition, while middle-aged adults constitute the bulk of the active population.

The horses were distributed into different age groups, with a predominance of horses aged 5–15 years, as expected because in some equestrian disciplines, as dressage and show jumping, the peak performance is generally reached between 12 and 16 years of age and the most reliable age for finishing endurance rides is between 10 and 15 years (27). This is a different situation from that of racehorses, whose peak performance age is 4.45 years (28).

The protocol used as a model for the present study was used by Romero et al. (13) to evaluate the welfare of pulling horses in work settings, and proved to be an easy, straightforward, and effective tool for assessing welfare in tiro y arrastre competitions, a context in which the competitive component may pose additional challenges to horse welfare by increasing workloads, physiological and behavioral arousal, and limiting opportunities for self-regulated pacing.

The environmental conditions, close to the threshold of heat stress and typical of the summer in the study area, when most competitions take place, indicate that, on certain days in August, horses may be at risk of heat stress due to high temperatures and humidity (26, 29), but none were dehydrated so horses seems to cope adequately to these conditions and they have access to water.

The owners and caretakers of the horses in this study were exclusively men (100%), and this result agrees with a previous study in working horses in Sudan (30) and exceed the published data of Italian Heavy Draft Horse (31) and working horses in Ethiopia (32) and contrasts with other equestrian disciplines where women play an important role. In fact, 70% of the equestrian licenses issued annually in Spain are granted to women (33). The reviewed literature indicates considerable consensus that women show greater concern for issues related to animal welfare (34). Furthermore, higher levels of handling by male humans may reduce horses’ social confidence while increasing compliance, suggesting that gender imbalances in human–horse interactions could negatively affect horse welfare by elevating stress in socially demanding situations (35).

The low proportion of horse owners with higher education can be attributed to the predominance of men engaged in agricultural activities, sectors in which vocational training is more common (36). Although one might assume that this lower level of formal education would correspond to reduced concern for horse welfare (34), the study demonstrates that these owners nonetheless provide a high level of care, dedicating substantial time to the training and maintenance of their animals.

Since the owners generally do not breed their own horses but rather acquire them at horse fairs in northern Spain, (Catalonia and Navarra) the horses competing in these events do not correspond to a homogeneous morphotype or genotype, nor are they the product of genetic selection programs aimed at sporting performance (3). Instead, horses of different breeds, and even crossbreeds, participate in these competitions. This contrasts with other equestrian disciplines, where genetic selection for sporting purposes (37, 38), along with morphological selection (39, 40), is a key factor. In the present case, the focus is placed primarily on the training of the horses, which is quite intensive, averaging nearly 4 days per week.

The information collected from the owners of draught horses indicates that these horses are generally highly valued and are an integral part of the culture and landscape of the region, also participating in cultural or religious events such as the Corpus Christi and festive parades in the Valencian Community (3).

The owners of the evaluated horses visited the veterinarian frequently, as shown in the survey, which demonstrates their concern, commitment, and positive healthcare toward their horses (20). However, other studies have reported that some draught horse owners fail to provide adequate healthcare for their horses (18, 30). The fact that participation in” tiro y arrastre” competitions is often a family tradition, combined with the high level of care and training that most owners provide to their horses, results in substantial knowledge of their animals’ health and welfare. This contrasts with riders of other types of sport horses, where such close, long-term involvement is less common (41). The horse’s participation in only 6–7 competitions per year contrasts with the very frequent training of almost 4 days a week, an intense dedication from the owner who undoubtedly takes great care of his horse’s physical fitness and health. The horse’s participation in only 6–7 competitions per year contrasts with the very frequent training of almost 4 days per week, reflecting the owner’s intense dedication to maintaining the horse’s physical fitness and health. These training frequencies are comparable to those recorded in high-level international competition horses in dressage and show jumping, which typically train 4–6 days per week. Furthermore, draft horses have longer training sessions, averaging 2.3 h, compared to sport horses, whose sessions typically range from 30 to 120 min (42–44).

### Behavioral indicators

The behavioral observations used in this study have previously been employed in other equine welfare research to assess an horse’s responsiveness to its environment, to identify fear or aggression toward humans (19), as well as to evaluate human–equine interactions (30, 45). Fear is considered a negative motivational state, as in horses it poses a risk of serious injury to owners or handlers. In this study, the most predominant behavioral response to the environment was an alert state, a finding consistent with other observational studies in draft horses (12, 13, 30).

From an animal welfare perspective, it is positive that most horses exhibited a general state of alertness, understood as a sensory attentiveness behavior involving the perception of visual, auditory, olfactory, and tactile stimuli. This response reflects the horse’s interest and willingness to actively interact with its environment (14). Conversely, 35% of the horses appeared apathetic, a state generally considered an indicator of compromised welfare and often associated with illness, fatigue, chronic pain, lethargy, depression, dehydration, or inconsistent handling (46, 47). However, given that very few horses were clinically ill in the present study, this apathetic demeanor may also reflect relaxation or habituation to the competition environment rather than poor welfare.

Most of our studied horses displayed friendly or affiliative behavior responses toward an unfamiliar observer that should be interpreted with caution and cannot be directly equated with the quality of the horse–owner relationship. Previous studies have demonstrated that horses are highly sensitive to human familiarity, attitude, trust, and behavior, and that these factors significantly influence their behavioral and physiological responses (48). In addition, horses can discriminate human emotional cues and respond differently to positive and negative expressions (49). Therefore, the friendliness or avoidance observed in the present study likely reflects the horses’ immediate appraisal of an unfamiliar observer and the interaction context, rather than their long-term relationship with their owners.

Similarly to our findings, Romero et al. (13) reported a high proportion of alert animals and predominantly friendly responses toward the observer, suggesting generally positive human–equine interactions. However, those authors also described a higher prevalence of apathetic or avoidant animals, which they associated with chronic workload, fatigue, or persistent discomfort. In contrast, the relatively low prevalence of apathy observed in tiro y arrastre horses may be explained by the short duration of effort, structured training, and adequate recovery periods characteristic of this discipline.

Within this framework, the behavioral responses observed may be interpreted as compatible with adequate welfare conditions. Friendly or affiliative behaviors are commonly associated with positive emotional states, reflecting comfort, security, and wellbeing. However, younger horses (<5 years) showed significantly more avoidance and aggressive responses toward the observer’s approach. Such responses are typical across species, as bonds with humans develop gradually through repeated positive interactions and trust-building during training (50), which in draft horses generally begins at approximately one year of age and continues until the third or fourth year (3). These behaviors are therefore more likely indicative of nervousness or heightened arousal rather than intentional aggression.

There was also a non-significant tendency for horses in categories 1 and 2 to exhibit avoidance or aggressive behaviors, which may be related to the predominant breeds in these groups—Jaca Navarra and Pottoka. These breeds are described as rustic, strong, spirited, and hot-blooded, with temperaments less selected for draft work (51, 52). Similar behavioral differences have been documented in working donkeys and mules (53).

Older horses were more likely to accept physical contact with the observer, supporting previous findings that age and cumulative positive interactions increase the acceptance of human contact or affiliative responses toward humans (54). Overall, while the observed friendly behaviors suggest generally positive welfare and human–horse relationships, responses toward unfamiliar observers reflect immediate emotional states, age, and breed characteristics, rather than the overall quality of the human–horse bond.

As older horses become habituated to the different stimuli involved in competition, they are less affected by novel situations, which is positive for their wellbeing. Younger horses have more innate fear and flight responses, since they have not yet been sufficiently exposed to novel stimuli with incomplete habituation processes (55).

The atmosphere of competitions involves novel stimuli (spectators, noises). A horse that trains 4 days a week, alone with its owner, is not used to interacting with other people, except in the six or seven competitions it participates in each year. For younger horses, the environment is even more novel and stressful due to their lack of experience.

The high frequency of training may contribute to the predominantly friendly responses observed, similar to patterns seen in working horses (4). Moreover, positive responses to observers may be related to the level of empathy owners have toward draft horses, which are often treated as companion animals. With an average of 2.4 horses per owner, close contact is promoted, and the structured training schedule of 4 days per week helps strengthen the bond with the trainer, as reported in other studies with draft horse owners (12, 42, 56, 57).

Other studies using similar tests in draft horses have reported different results, with higher rates of aggressive behavior reflected in avoidance responses (14, 58, 59). These differences may be influenced by horses’ cognitive ability to recognize familiar humans (50, 60), including facial recognition (61), or even to recall past negative experiences (5). Given that the observer in this study was unfamiliar to the horses, it is reasonable to expect different reactions depending on the horse’s prior experience with the person conducting the test (13). Negative prior human–equine interactions can lead to exaggerated fear responses (62). In our case, excessive fear could limit a horse’s participation in competitions by making them dangerous to handle.

Despite the considerable amount of research focused on welfare in sport horses, understanding how to assess the emotions of competition horses can guide owners and caregivers in identifying practices and activities that should be encouraged, avoided, or even prohibited in the horse’s life (63). Accordingly, the development and validation of standardized behavioral indicators, such as those used in this study, can contribute significantly to improving equine management and welfare (64, 65).

### Health indicators

Body condition scoring in horses is considered a highly useful and relevant welfare indicator, as it relates to conditions such as malnutrition, overfeeding, metabolic disorders, or laminitis, among others (66). Romero et al. (13) also reported that the majority of working equids showed an acceptable body condition score; however, they found significant associations between low body condition and the presence of skin lesions and systemic health problems. Such associations were not observed in the present study, which may reflect the lower cumulative workload and better access to preventive veterinary care in competitive draft horses compared to working equids subjected to daily labor.

In our study, no horse exhibited body condition scores outside the normal range, with scores ranging from 2 to 4 out of 5, and the majority being at score 3. This is noteworthy, as one might expect some owners to underfeed horses to compete in lower-weight categories. No significant differences were found between the body condition of different weight categories or age groups. Similarly, no signs of dehydration were observed in any horse, which is also relevant because some horses could weigh less if deprived of water prior to competition to participate in lower-weight classes.

In previous studies assessing draft horse welfare, cutaneous lesions were frequently reported (13, 30, 67, 68), with higher prevalence in horses older than 5 years. Similarly, in our study, lesions were detected at headstall points (15.63%) and at the neck and chest where the harness is placed (8.13%), results in accordance with Adam et al. (30), with incidence significantly higher in older horses (*p* < 0.001), likely resulting from cumulative wear from training and competition over time. Nevertheless, these lesions were very mild: small, superficial alopecic scars that did not affect subdermal tissue. The incidence rate is considerably lower than in other studies where skin lesions affected more than 26% of horses and involved deeper tissues (13), rising 44% in the study of Chaburte et al. (67) indicating that the low lesion rate in our study reflects effective preventive management by the owners rather than absence of risk.

Harness-related skin lesions are a major welfare concern in working equids. Romero et al. (13) reported a high prevalence of lesions affecting the head, withers, spine, and ribs, in some cases involving deeper tissues, likely related to prolonged use of poorly fitted equipment. In the present study, only some mild harness and headstall related lesions were observed. This difference may be attributed to the regulated nature of “tiro y arrastre” competitions, the shorter exposure time to harness pressure, and the generally adequate adjustment of equipment.

Lesions on the neck, chest, and withers are associated with the quality of the harness and collar, which can cause significant pain, particularly in the neck, and may impair the horse’s ability to carry weight, especially when the load is heavy, as is typical in draft and pulling competitions (69, 70).

Hoof condition is an important welfare indicator in horses, reflecting the care provided by the owner, particularly in sport horses, as poor hoof condition is a common cause of lameness (63). In this study, abnormal hoof quality was rare (6.25%) with much lower values than reported previously in working horses (12, 30). However, overgrown or poorly maintained hooves were observed at a relatively high rate for sport horses (19.38%), which could create dynamic imbalances due to uneven pressure on the hoof biomechanics, potentially causing ligament or tendon strain and altering gait (30, 71, 72). No such gait alterations were observed, likely because overgrowth was mild in all cases and the competition surface was soft dirt, which may mask mobility issues. Most sport draft horses are shod. Although they train and compete on arena surfaces and many owners believe that traction may be better without horseshoes, these horses frequently travel on paved roads from their stables to the training grounds, often on an almost daily basis. This regular exposure to hard surfaces results in considerable hoof wear, making shoeing necessary to protect hoof integrity and maintain soundness.

The observed hoof care deficiencies may be related to improper training management by the owner, economic limitations that reduce farrier visits, or lack of knowledge about proper hoof maintenance practices (73).

Observed conformation defects may result from minor congenital morphological issues, which are more common due to the absence of selective breeding for sport use (3). These defects do not appear to affect mobility or, likely, athletic performance, given the specific demands of this equestrian discipline.

The mild fever detected in two horses, along with the high percentage of horses with increased heart rate (HR) and respiratory rate (RR), suggests that environmental heat and stress from pre-competition handling or post-transport may contribute to nervousness in horses before the sporting event (74). The significantly higher HR and RR in younger horses (*p* < 0.01) supports this hypothesis, as older, more experienced horses tend to remain calmer, consistent with the behavioral patterns observed, and therefore exhibit lower HR and RR.

Considering the previously reported results we recommend that the Federation and owners enhance training in preventive hoof care and proper harness use, implement regular veterinary check-ups during competitions, and develop awareness programs emphasizing the importance of rest and recovery periods.

### Relationship between behavioral and health indicators

The significant correlation between the aggregated behavior score, general attitude and response to the observer’s approach/passage, and heart rate (HR) and respiratory rate (RR) (Table 8) suggests that horses experience a state of arousal during rest periods, either after being unloaded from the truck or just before the competition. This heightened state may alter the horse’s basal metabolism, increasing alertness and reactivity toward people nearby. Unlike other equestrian disciplines, where horses remain in their stables for several days and compete close by, in draft horse pulling competitions the horses travel to the venue on the same day, with transport lasting between 30 min and 2 h. Only a few hours are available for acclimatization before competition, making it possible for the horses to experience a certain level of “pre-competition stress.”

**Table 8:** Frequency (%) and total number (*n*) of the animal welfare indicators assessed in the evaluated horses (*n* = 160).

		Category					Age			*p*-value*	*p*-value*	Total
		1	2	3	4	5	<5	5–15	>15			
*n*		27	25	41	35	32	24	92	44	Cat.	Age	
Body condition	2	14.81 (4)	8.00 (2)	9.76 (4)	5.71 (2)	0	8.33 (2)	8.70 (8)	4.55 (2)	0.14	0.0652	7.50 (12)
	3	77.78 (21)	80.00 (29)	73.17 (30)	77.14 (27)	65.63 (21)	54.17 (13)	75.00 (69)	84.09 (37)			74.38 (119)
	4	7.41 (2)	12.00 (3)	17.07 (7)	17.14 (6)	34.38 (11)	37.50 (9)	16.30 (15)	11.36 (5)			18.13 (29)
Lesions at headstall contact points		22.22 (6)	16.00 (4)	17.07 (7)	8.57 (3)	15.63 (5)	0	5.43 (5)^a^	45.45 (20)^b^	0.75	<0.001	15.63 (25)
Neck and chest lesions at harness contact points		7.41 (2)	4.00 (1)	7.32 (3)	14.29 (5)	6.25 (2)	0	3.26 (3)^a^	22.73 (10)^b^	0.67	<0.001	8.13 (13)
Limb alignment deviations		11.11 (3)	20.00 (5)	9.76 (4)	5.71 (2)	15.63 (5)	12.50 (3)	11.96 (11)	11.36 (5)	0.55	0.99	11.88 (19)
Joint inflammation		3.70 (1)	0	4.88 (2)	0	0	0	3.26 (3)	0	0.38	0.33	1.88 (3)
Poor hoof quality		7.41 (2)	4.00 (1)	7.32 (3)	5.71 (2)	6.25 (2)	0	6.52 (6)	9.09 (4)	0.99	0.35	6.25 (10)
Excessive length of hoof wall		29.63 (8)	24.00 (6)	12.20 (5)	25.71 (9)	9.38 (3)	25.00 (6)	20.65 (19)	13.64 (6)	0.27	0.54	19.38 (31)
Abnormal temperature (>38.5°C)		0	0	2.44 (1)	0	3.13 (1)	0	2.17 (2)	0	0.65	0.48	1.25
Altered resting heart rate (>48 ppm)		70.37 (19)	60.00 (15)	43.90 (18)	62.86 (22)	78.13 (25)	83.33 (20)^a^	70.65 (65)^a^	31.82 (14)^b^	0.43	<0.01	61.88 (99)
Altered resting respiratory rate (>20 rpm)		37.04 (10)	32.00 (8)	26.83 (11)	17.14 (6)	28.13 (9)	83.33 (20)^a^	70.65 (65)^a^	58.33 (14)^a^	22.83 (21)^b^	20.45 (9)^b^	0.65	<0.01	27.5 (44)

Groups with different letters in red show significant differences (*p* < 0.05, post-hoc test).

This contrasts with situations reported in some studies where draft horses display a lack of alertness or responsiveness to observers, and even depression (30), often linked to health problems or physical exhaustion that result in behavioral unresponsiveness (75). Such scenarios, commonly observed in countries where draft horses are primarily used as working animals, are very different from our sporting context.

Skin lesions caused by harnesses and collars were correlated with the aggregated score of limb defects. Although these lesions were mild, they reflected some degree of neglect, mainly in hoof care, suggesting that better management and attention to the horses could reduce these minor injuries, which should ideally be prevented.

Monitoring HR, RR, and body temperature (Table 9) revealed large, significant increases over a short period (mean 65 s), with HRs similar to those observed in equestrian disciplines such as show jumping (170.8 bpm) (76), but lower than the peak HRs recorded in trotting championships (207 bpm) (77). Reaching these bpm levels represents maximal physical exertion for a horse, so a rise in body temperature of about 1 °C after the test is expected. The fact that the horses returned to resting values after 15 min of recovery (Figure 1) and that hematological analysis was entirely normal (Table 6) indicates that these horses are well-trained for the competition and fully capable of handling the physical demands of the event.

**Table 9:** Correlations between aggregated behavioral scores and analyzed health parameters.

Behavioral and health parameters		Correlation (*r*)	*p*-value
Horse reactivity to observer^1^	Body condition	0.13	0.17
	Skin lesions	−0.07	0.36
	Limb problems	0.09	0.12
	Heart rate	0.22	<0.05
	Respiratory rate	0.11	<0.05
Body condition	Skin lesions	−0.18	0.14
	Limb problems	0.05	0.11
	Heart rate	0.14	0.10
	Respiratory rate	0.11	0.18
Skin lesions^2^	Limb problems	0.12	<0.05
	Heart rate	0.24	0.22
	Respiratory rate	0.19	0.28
Limb problems^3^	Heart rate	0.09	0.13
	Respiratory rate	0.12	0.25
Heart rate	Respiratory rate	0.21	<0.05

Red values indicate *p* < 0.05.

1 Aggregated score “horse reactivity to the observer”: general attitude + response to observer approach + response to observer walking alongside.

2 Aggregated score “skin lesions”: harness-related lesions on head and collar-related lesions on neck and chest.

3 Aggregated score “limb problems”: conformation defects, joint inflammation, poor hoof quality, and hoof overgrowth.

**Figure 2:**
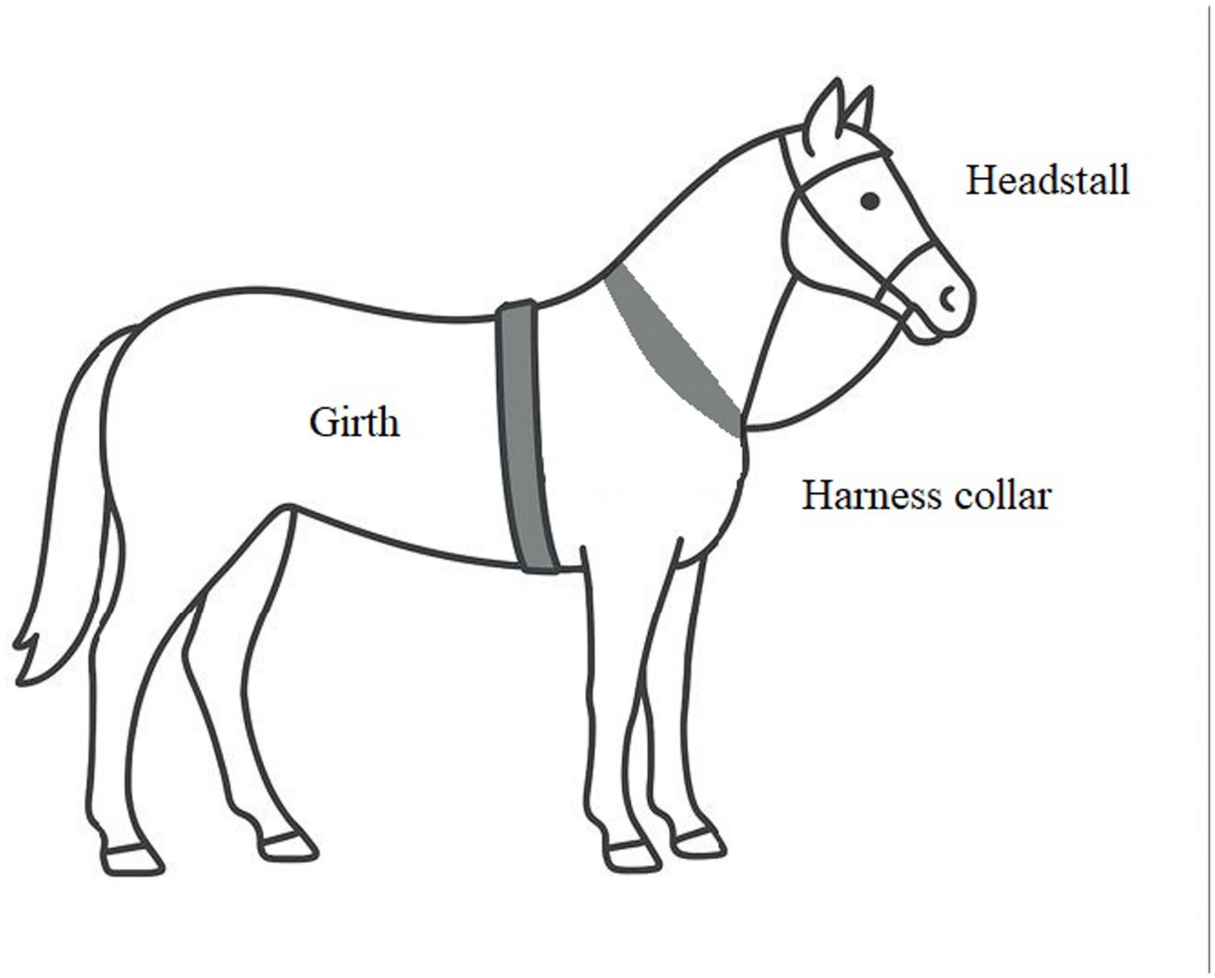
Body contact points with harnesses, headstall, and girth on the draft horse.

Overall, these findings emphasize the importance of interpreting welfare assessments within the specific context of the type, duration, and intensity of work performed by equids. Although horses subjected to continuous daily labor may experience cumulative welfare challenges (13), the results of the present study indicate that “tiro y arrastre” horses, despite undertaking physically demanding tasks, do not exhibit major welfare impairments when appropriate training, management, and recovery are ensured. This underlines the critical role of adequate husbandry and workload management in maintaining acceptable welfare standards in working horses and supports the need for context-sensitive welfare evaluations (Table 9 and Figure 2).

## Conclusion

This study provides the first comprehensive description of welfare in horses participating in Spanish draft competitions. Overall, horses exhibited good health, adequate body condition, and positive human–horse interactions, reflecting appropriate management practices.

Several key risk factors were identified. Mild harness-related lesions and hoof overgrowth indicate areas where preventive management should be strengthened. Behavioral signs of apathy—particularly in younger and smaller horses—suggest that training intensity and workload should be adjusted according to age and body size.

Based on these findings, several evidence-based recommendations can be proposed to safeguard horse welfare in this discipline: (1) the implementation of regular preventive hoof-care routines; (2) systematic monitoring and adjustment of harness fit; (3) progressive, category-specific training programs for inexperienced or younger horses; and (4) routine veterinary check-ups during competitions, together with awareness initiatives on rest and recovery.

Overall, physiological and hematological responses confirmed adequate conditioning, indicating that participation in draft competitions, under current management practices, does not significantly compromise welfare. Nonetheless, adopting the proposed measures would help minimize identified risks and promote sustained welfare standards within this traditional equestrian sport.

In addition to providing practical recommendations, the patterns observed in this study constitute initial evidence on welfare-related interactions, helping to shape future research questions and hypothesis-driven investigations in this traditional sport.

## Data Availability

The raw data supporting the conclusions of this article will be made available by the authors without undue reservation.

## Appendix 1: questionnaire completed with the owners of the horses studied

1. Owner-related information

    ○  Age and sex
    ○  Educational level (primary, secondary, university)
    ○  Place of residence (urban or rural)

2. Horse ownership and management

    ○  Number of horses owned
    ○  Location of the stable (same municipality or different municipality)
    ○  Source of horse acquisition (own breeding, another owner, horse fairs)
    ○  Age of the horse at purchase

3. Housing and daily management

    ○  Housing system (individual, social group and how many horses together)
    ○  Access to outdoor paddocks (yes or not)
    ○  Availability of indoor stalls (yes or not)
    ○  Type of diet (pasture-based diet, roughage-based diet, concentrate-rich diet, mixed diet)

4. Training practices

    ○  Age at which training started
    ○  Training frequency (days per week)
    ○  Training duration (hours per session)
    ○  Person responsible for training and daily care (owner, trainer, or shared)

5. Competition-related information

    ○  Number of competitions participated in per year
    ○  Motivation for participation (e.g., family tradition or personal interest)

6. Veterinary care and health management

    ○  Frequency of veterinary visits per year
    ○  Horse identification and basic data
    ○  Horse age
    ○  Breed
    ○  Sex
    ○  Identification via RFID tagging

All owners were informed about the objectives of the study and provided written informed consent. The information collected was treated anonymously.
